# Investigation of 29 Antimicrobial Compounds in Soil Using Newly Developed UHPLC-MS/MS Method

**DOI:** 10.3390/molecules28186496

**Published:** 2023-09-07

**Authors:** Małgorzata Gbylik-Sikorska, Anna Gajda, Monica Felipe-Sotelo, Manuela Caniça, Adriana Cabal-Rosel, Tanel Tenson, Marta Kořínková, Krõõt Arbo, Veljo Kisand, Gerhard Rab, Martin Brandtner

**Affiliations:** 1 Department of Pharmacology and Toxicology, National Veterinary Research Institute (NVRI), Al. Partyzantow 57, 24-100 Pulawy, Poland; anna.gajda@piwet.pulawy.pl; 2 Faculty of Engineering and Physical Sciences, School of Chemistry and Chemical Engineering, University of Surrey, Guildford GU2 7XH, UK; m.felipe-sotelo@surrey.ac.uk; 3 National Reference Laboratory of Antibiotic Resistances and Healthcare Associated Infections, Department of Infectious Diseases, National Institute of Health Dr. Ricardo Jorge (INSA), 1649-016 Lisbon, Portugal; manuela.canica@insa.min-saude.pt; 4 Centre for the Studies of Animal Science, Institute of Agrarian and Agri-Food Sciences and Technologies, University of Porto, 4050-453 Porto, Portugal; 5 Associate Laboratory for Animal and Veterinary Sciences, AL4AnimalS, 1300-477 Lisbon, Portugal; 6 CIISA—Center for Interdisciplinary Research in Animal Health, Faculty of Veterinary Medicine, University of Lisbon, 1300-477 Lisbon, Portugal; 7 Austrian Agency for Health and Food Safety (AGES), 1220 Vienna, Austria; adriana.cabal-rosel@ages.at (A.C.-R.); martin.brandtner@ages.at (M.B.); 8 Institute of Technology, University of Tartu (UT), 50411 Tartu, Estonia; tanel.tenson@ut.ee (T.T.); kroot.arbo@ut.ee (K.A.); kisand@ut.ee (V.K.); 9 National Institute of Public Health—(NIPH), 10042 Prague, Czech Republic; marta.korinkova@szu.cz; 10 Institute for Land and Water Management Research, Federal Agency for Water Management, 3252 Petzenkirchen, Austria; rab@hydro.tuwien.ac.at

**Keywords:** antimicrobial agents, UHPLC-MS/MS, soil, environment

## Abstract

While the prudent and reasonable use of veterinary antimicrobial agents in food-producing animals is necessary, researchers over the decades have shown that these antimicrobial agents can spread into the environment through livestock manure and wastewater. The analysis of the occurrence of antimicrobial compounds in soil samples is of a great importance to determine potential impacts on human and animal health and the environment. In this study, an affordable, rugged and simple analytical method has been developed for the determination of twenty-nine antimicrobial compounds from five different classes (tetracyclines, fluoro(quinolones), macrolides, sulfonamides and diaminopirimidines). Liquid–liquid extraction (LLE) with extract filtration combined with ultra-high performance liquid chromatography tandem mass spectrometry (UHPLC-MS/MS) was the best strategy for the simultaneous determination of all analytes. The developed method was validated according to the Commission Implementing Regulation (EU) 2021/808. The limit of detections (LODs) ranged from 0.5 to 2.0 µg/kg, while the limit of quantitation (LOQ) was established at 1.0 to 20.0 µg/kg. The developed method was successfully applied for the determination of antimicrobial residues in one hundred and eighteen soil samples obtained from four European countries (Austria, Czech Republic, Estonia and Portugal). Doxycycline in the concentration levels of 9.07 µg/kg–20.6 µg/kg was detected in eight of the analysed samples. Samples were collected from areas where natural fertilizers (swine or cow manure) were applied. Our method can be efficiently used to monitor anti-microbial compounds in soil samples.

## 1. Introduction

Antimicrobial compounds, used with prudence, can support health and save life, but when overused or used without caution, can pose a significant threat. The seventh Annual Report on Antimicrobial Agents Intended for Use in Animals presents data analyses with a special focus on the antimicrobial quantities reported to be used in 2019 by 110 participant countries around the world [1]. Considering sales and import data, the World Organisation for Animal Health (WOAH) estimates that a total of 77,086 tonnes of antimicrobial agents intended for animals were used in 2019. The WOAH also estimated that in 2019 a total of 99.09 to 108.49 milligrams of antimicrobial agents were used per kilogram of animal biomass, depending on how coverage estimates were adjusted among the 108 participants [1]. The twelfth report of The European Surveillance of Veterinary Antimicrobial Consumption (ESVAC) presents sales of veterinary antimicrobial agents in 31 European countries in 2021 and trends in sales between 2010 and 2021 in 25 countries [2]. The indicator of sales of antibiotic veterinary medicinal products (VMPs) for food-producing animals used in the ESVAC report is the milligrams (mg) of active substance sold per population correction unit (PCU)—mg/PCU. Considering data collected from 2010 to 2021, the sales of antibiotic drugs declined from 161.2 to 86.2 mg/PCU for 25 countries. The sale of antibiotic VMPs marketed mainly for food-producing animals in 2021 reported for 31 European countries was 84.4 mg/PCU. In addition, the total sales of AMEG Category B antibiotics have decreased since 2011 in 25 countries. Sales of third- and fourth-generation cephalosporins decreased from 0.24 to 0.15 mg/PCU for fluoroquinolones, other quinolones from 2.5 to 2.2 mg/PCU and from 1.1 to 0.18 mg/PCU, respectively, and for polymyxins, from 11.0 to 2.2 mg/PCU. Tetracyclines, penicillins and sulfonamides were the top three highest-selling antibiotic classes between 2011 and 2021. Also, the WOAH’s annual seventh Report on Antimicrobial Agents Intended for Use in Animals shows that tetracyclines are the most utilised antimicrobial class globally in animal health [1]. The data indicated that the use of antibiotics is declining in most countries of the world, and this trend is particularly pronounced in European countries. Unfortunately, the amount of antibiotic use is still high and it is necessary to conduct research, including environmental studies, to monitor the consequences.

Although sales have declined in most European countries, antimicrobial compounds are and will be used to treat bacterial infections in food-producing animals. Up to 90% of veterinary antimicrobial agents may be excreted as parent compounds and/or metabolites [3,4]. The production of waste, such as animal faeces, urine and manure, in the European Union’s 27 countries from 2014 to 2018 stayed at almost 10 million tonnes per year [5]. The utilisation of waste from the animal husbandry industry is crucial from an environmental, economic, human and animal safety point of view. Animal by-products (ABPs), such as manure (animal faeces, urine and plant material), which belong to category 2 in accordance with Regulation (Ec) No 1069/2009 of the European Parliament and of the Council of 21 October 2009 may be applied to land without processing as a natural fertilizer [6].

The high content of nutrients of solely biological origin: carbon, nitrogen, potassium and phosphorus has made these fertilisers very attractive to the agricultural industry [7,8]. As was previously described, manure-based organic fertilisers may be contaminated with VMPs, including antimicrobial agents and/or their metabolites [3,9,10,11]. Many authors report soil contamination with antimicrobial compounds caused by the use of organic manure-based fertilisers or wastewater from animal husbandry [3,4,10,11,12,13,14,15]. Moreover, we can also find researches describing crop contamination with antibiotics caused by the use of organic manure-based fertilisers [11,16,17,18,19]. The accumulation and persistence of antibiotics in soil depends on a variety of factors such as soil type, moisture content, sorption and the physical and chemical properties of the substances and their stability [20]. Fluoroquinolones can be found in the soil even for many years. Other results show that tetracyclines have been determined in soil from 5 months after manure application [21] to several years [22]. Sulfonamides can be found in soil samples for a few days, while macrolides showing half-lives up to few months and β-lactam antibiotics were degraded in a few days [20].

The presence of antimicrobial agents in soil affects its microorganisms, and even in very low concentrations, contributes to the genetic changes in bacterial genomes and the increased environmental burden of antibiotic resistance genes (ARGs) [15,20,23,24,25,26,27]. Antimicrobial resistance (AMR) poses a significant global threat to human, animal and environmental health and is one of the target One Health research areas [28,29,30]. The transmission routes of ARGs between humans and the environment, food animals and the environment and finally between food animals and humans are poorly recognized. The possibility of increasing our knowledge of soil contamination by veterinary antimicrobials in different countries and agricultural areas has an impact on the recognition of this problem.

Several multi-component methods are available in the literature for the determination of various veterinary antimicrobial drugs in soil [3,10,11,12,13,14,31,32,33,34,35,36,37,38]. Most of them enable the determination of only one class of compounds [14,37] or one substance from different xenobiotic classes [38] or up to four different classes [10,11,12,13,32,33]; only a few of them enable the determination of many substances from different classes [35,36]. Frequently, the isolation of analytes is based on liquid–solid extraction (LSE) coupled with solid-phase extraction (SPE) using one [3,12,13,14,32,38] or a combination of two different cartridges [10,35]. Most of the methods are based on LC-MS/MS (liquid chromatography-tandem mass spectrometry) analysis [3,10,13,35,38].

Our study aimed to develop a new affordable, low-time consuming and low-cost multi-class and multi-component analytical method for the determination of twenty-nine antimicrobial compounds from five classes in soil samples. In addition, the developed and fully validated method was applied to investigate the occurrence of antimicrobial drugs in 118 soil samples taken from four different European countries. The obtained results will be useful for assessing the environmental safety of agricultural soil.

## 2. Results and Discussion

### 2.1. Optimization of LC-MS/MS Conditions

Based on previously published papers, electrospray ionization (ESI+) in a positive mode was investigated in mass spectrometric analysis under the multiple reaction monitoring (MRM) mode to achieve the most abundant signal for each analyte [3,13,32,38,39]. For the target compounds, two transition products were monitored; for internal standards (IS), one transition was selected to improve quantitation and confirmation of the development method. The MRM transitions and mass source parameters are presented in Table 1.

**Figure 1:**
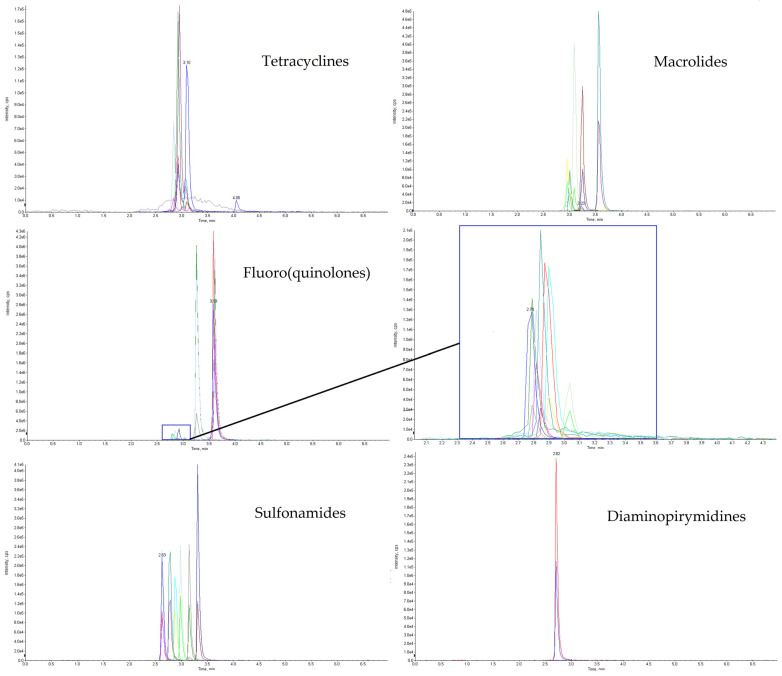
Chromatogram of soil sample spiked with a mixture of 29 analytes at a 20 µg/kg concentration level.

During the development of the method, a few combinations of aqueous and organic mobile phases were tested, including formic acid and heptafluorobutyric acid, HFBA (as an ion -pair agent) at various concentrations (0.25–5% and 0.025–0.1%, respectively), acetonitrile, methanol and acetonitrile with methanol (90:10, 80:20, 20:80 and 10:90, *v*/*v*). With the purpose of achieving the best chromatographic separation in the shortest possible analysis time, four different HPLC columns were evaluated, namely Phenomenex Luna C18 (2) 100 A column (50 mm × 3.0, 3 µm) and Agilent ZORBAX SB-C18 column (50 mm × 2.1 mm, 1.8 µm), in various gradient elution programs at different flow rates. All of the tested HPLC columns were suitable for antimicrobial compounds chromatographic separation, but the optimal results considering the shape of the peaks and the resolution of the analytes were accomplished with a Phenomenex Luna^®^ Omega Polar C18 10 column, Phenomenex, Torrance, CA, USA (100 × 2.1 mm, 1.6 µm) and Agilent InfinityLab Poroshell 120 EC-C18, Agilent Technologies, Santa Clara, CA, USA (2.1 × 150mm, 2.3 µm). Finally, the best results were achieved using 0.025% HFBA (B) and acetonitrile (A), a gradient started with 92% of B and 8% of A mobile phase and Luna^®^ Omega Polar C18 10. For the selected column, the analysis time was shorter at relatively low flow rates (0.4 mL/min) compared with the Agilent InfinityLab Poroshell 120 EC-C18 column for which the same time of analysis could only be achieved at high flow rates (>0.6mL/min), which would shorten its life significantly. The extracted ion chromatograms (XICs) of the five different antimicrobial compounds group are presented in Figure 1.

### 2.2. Optimization of Sample Preparation

One of main goals of the present study was to develop a fast and low-cost sample preparation procedure. To account for the influence of factors such as soil particle size and water content, the sample pretreatment step was carefully optimized. Different conditions of drying were tested: room temperature and incubation at 20–35 °C for 24 h. The best results were achieved by drying the sample at 25–30 °C/24 h in a laboratory incubator. In order to minimize the differences in soil grinding conditions, three metal sieves with different mesh diameters (0.5; 0.8 and 1.2 mm) were checked. Finally, we chose a sieve with a mesh diameter of 0.5 mm.

**Figure 2:**
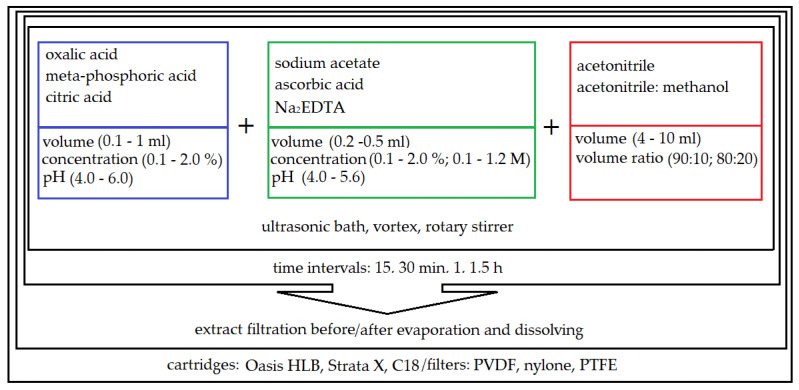
Optimization of the multi-residue extraction of antimicrobials from soils samples using different liquid–solid extraction (LSE) procedures.

Different variants of liquid–solid extraction (LSE) were tested, assisted by an ultrasound, vortex or rotary stirrer, coupled with different filtration techniques (syringe filter and SPE cartridges). Based on previous experience in developing multi-compound methods in variety of matrices (e.g., in sediments) [39], it was decided to test different organic solvents with the addition of acids in the extraction step. A schematic of the reagents tested in the sample preparation optimization process is shown in Figure 2.

**Figure 3:**
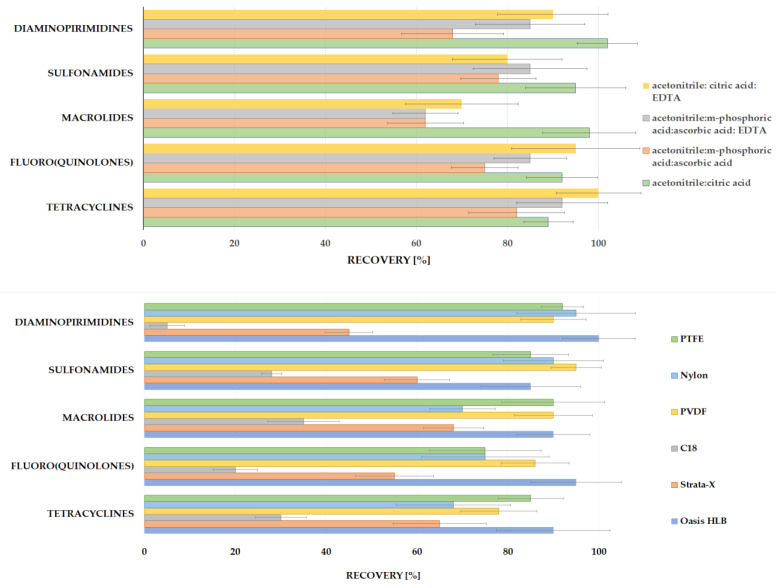
The comparison of average recoveries (*n* = 3) obtained using the most optimal different extraction mixtures and different methods of extract filtration.

Various extraction mixtures were then investigated to obtain the best isolation of five different classes of compounds from soil samples. As is shown in Figure 2, a wide variation of solvents with the addition of acids and the salts of acids and the order of addition of individual reagents were tested. The optimal recoveries of the analytes >60% were obtained for the mixture of acetonitrile followed by the addition of citric acid; acetonitrile followed by the addition of citric acid and Na_2_EDTA; acetonitrile followed by the addition of meta-phosphoric acid and ascorbic acid; acetonitrile followed by the addition of meta-phosphoric acid, ascorbic acid and Na_2_EDTA disodium salt dihydrate (Figure 3). The Na_2_EDTA disodium salt dihydrate was used to improve the recovery of tetracyclines or fluoro (quinolones) which may form chelates with metal ions e.g., Ca^2+^ and Mg^2+^ [40,41], present in the soil. The use of Na_2_EDTA disodium salt dihydrate enhanced the recovery for both groups, but significantly reduced it for selected macrolides (josamycin and azitromycin). This may be because Na_2_EDTA also chelates organic compounds, which can affect macrolide recovery. Moreover, the addition of 1 mL of ultrapure water to the dry soil sample is required to enable the isolation of analytes due to the hydrophilic characteristics of most compounds except sulfonamides. A comparison of average recoveries (measured for three replicates) for five various analyte classes according to the four selected extraction mixtures is shown in Figure 3. The addition of 10% citric acid pH = 4.0 allowed for very good recoveries (≥80%) for both tetracyclines and all macrolides. The use of citric acid pH = 4.0 makes it possible to obtain the optimal pH for both groups, that is, it is acidic enough to improve the isolation of tetracyclines without interfering with the isolation of macrolides, which degrade in an acidic environment.

In the next step, the extraction efficiency was selected by testing the usage of a vortex, ultrasonic bath and rotary stirrer in different time intervals and various configurations. The best extraction yield (recovery > 80%) was reached through a combination of different techniques (vortex, ultrasonic bath and rotary stirrer).

The purification of the extract was tested in two variants: filtration before evaporation (with C18, Strrata X and Oasis HLB as a filter) and filtration (PTFE, Nylon and PVDF) after evaporation and the dissolution of the dry residue. The best results (recovery > 80%) for all analytes were performed with the usage of SPE cartridge Oasis HLB as a filter, without a conditioning step, which helped in the reduction of some interfering components derived from soil. The worst results were obtained with C18 cartridges; the average recovery for all compounds was below 40% (Figure 3).

### 2.3. Method Validation

Method validation was performed according to the Commission Implementing Regulation (EU) 2021/808 of 22 March 2021 document [42] but without a decision limit (CCα) and detection capability (CCβ). The matrix-matched calibration curve showed good linearity in the range of LOQ—300 µg/kg, and the determination coefficients (r^2^) were found to be ≥0.997 for all compounds. The precision (within-laboratory reproducibility and repeatability) results obtained for LOQ validation level are summarised in Table 2. Both parameters were satisfactory for each analyte; the CVs for the % of repeatability and the within-laboratory reproducibility were lower than 15%. The average within-laboratory reproducibility and average CVs of repeatability were in the range of 1.4–14.9% and 5.0–15.0%, respectively. The limits of detections (LODs) were calculated in the range of 0.5 to 2.0 µg/kg, depending on the analyte; the limits of quantitations (LOQs) of the method were set as the lowest point of the calibration curve (5.0, 10.0 and 20.0 µg/kg), depending on the compound. The LOQ and LOD values are presented in Table 2. There were no significant interfering peaks in the blank soil samples (obtained from various regions and sorts) at the corresponding retention time of target analytes and ISs. This indicated that the selectivity of the presented method is suitable for the quantitation of all 29 compounds.

Table 2 shows that the recoveries obtained for the LOQ validation level of all the target antimicrobial drugs were in the range of 89–113%. The average recovery was in the range of 77–119%. The developed method is rugged because none of the four tested factors affected the reproducibility of the method. The presence of signal enhancement or suppression for the target compounds was regarded as matrix effect (ME, %). The MEs were lower than ±20% and were in the range of 83–118%, depending on the analyte. The value between 85% to 115% was considered not to be affected by matrix effects. As shown in Table 2, the ME was not observed for most compounds of interest. Both ion suppression and ion enhancement were observed for two analytes (josamycin and spiramycin). Therefore, the matrix match calibration curves were utilised to minimize and avoid the matrix effect.

### 2.4. Soil Samples Analysis

The proposed method was applied to determine 29 antimicrobial compounds in agricultural soil samples. In eight of the analysed samples, doxycycline in the concentration level of 9.07–20.6 µg/kg was detected. The samples in which the presence of an antimicrobial agent was detected were collected in Autumn before and after harvesting. In four samples, the soil was previously fertilized with manure, while in another four samples, an artificial fertilizer was used. Previously published papers also report the detection of tetracyclines in the range of 7–250 µg/kg [10,12,13], including doxycycline (0.1–500 µg/kg) [3,35], in soil samples. No antimicrobial drugs were detected in the other samples at concentrations above the LOD of the developed method. The obtained results confirmed the widespread use of doxycycline in animal husbandry. This antibiotic has a wide spectrum of activity against Gram-negative and Gram-positive bacteria, mycoplasmas, spirochaetes, rickettsias, chlamydias, and some protozoa [43,44]. The Antimicrobial Advice Ad Hoc Expert Group (AMEG) has categorised tetracyclines in category D (prudence), which means that they should be used as first line treatments, whenever possible, but only when they are medically inferior [45]. It is also worth mentioning that according to the WHO CIA list (Criticaly Importanat Antimicrobialsa for Human Medicine), tetracyclines are categorised as a highly important antimicrobial criterion C1, to treat serious bacterial infections in people [46].

Further research on the presence of antibacterial drugs and other xenobiotics, heavy metals and pesticides in soil is needed. Expanding the scope of research to include other matrices such as water, wastewater or manure and increasing the number of countries sampled would be important to expand knowledge in this area.

## 3. Materials and Methods

### 3.1. Samples Collection

**Figure 4:**
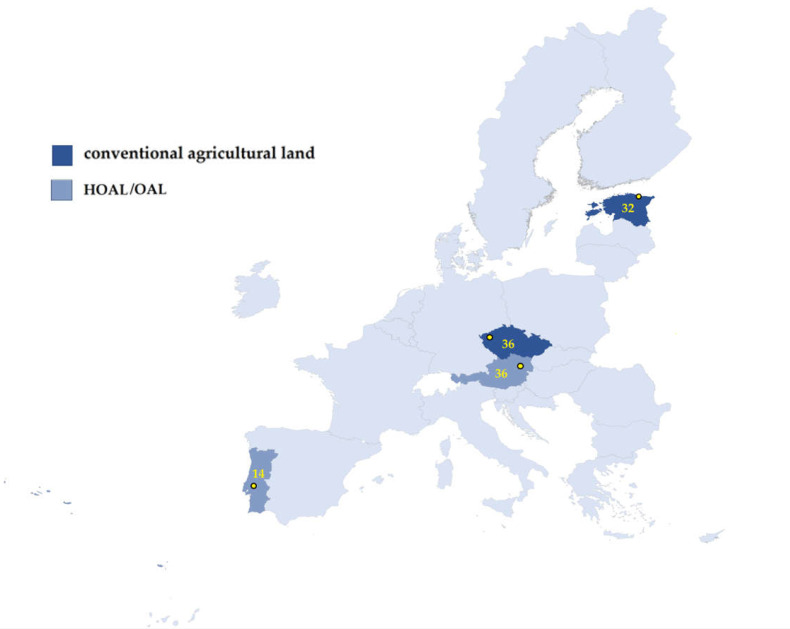
Map of the sampling point and number of collected soil samples.

The soil samples were collected as part of the project FED-AMR “The role of free extracellular DNA in the dissemination of antimicrobial resistance over ecosystem boundaries along the food/feed chain”—Research Project of the One Health European Joint Programme (OHEJP). Samples (118) were delivered from four European countries: Austria, Czech Republic, Estonia and Portugal (Figure 4). Samples were obtained from a variety of sources such as Hydrological Open Air Laboratories (HOALs) or Open Air Laboratory (OAL), or conventional agricultural land, as is shown in Table 3. Sampling was provided at four different time points: Spring, Summer, Autumn and Winter.

### 3.2. Chemical and Reagents

Acetonitrile and methanol were obtained in LC-MS grade (J.T. Baker, Deventer, the Netherlands); citric acid (purity 99.4%) and acetic acid (purity 99.5–99.9%) were purchased from POCH (Gliwice, Poland); and heptafluorobutyric acid (HFBA) was obtained from Sigma–Aldrich, (St. Louis, MO, USA). Oasis HLB 3 cc Vac Cartridge, 60 mg Sorbent per Cartridge (Waters, Milford, MA, USA). Milli-Q water was prepared using a Milli-Q Gradient Water System (Millipore, Molsheim, France; >18 MX cm^−1^).

The antimicrobial compounds oxytetracycline (OTC), tetracycline (TC), chlortetracycline (CTC), doxycycline (DC), demeclocycline (DMC), ciprofloxacin (CIP), enrofloxacin (ENR), difloxacin (DIF), danofloxacin (DAN), flumequine (FLU), marbofloxacin (MAR), sarafloxacin (SAR), norfloxacin (NOR), oxolinic acid (OXO), nalidixic acid (NAL), ciprofloxacin-d8 (CIP-d8), erythromycin (ERY), tylosin (TYL), tulathromycin (TLM), tilmicosin (TIL), josamycin (JOS) spiramycin (SPI), azytromycin (AZY), sulfamerazine (SME), sulfamethazine (SMT), sulfadimethoxine (SDMX), sulfamethoxazole (SMA), sulfamonomethoxine (SMM), sulfathiazole (SFT), sulfadoxine (SDX), sulfadiazine (SDZ), sulfafenazole (SFF), trimethoprim (TMP) and trimethoprim-d9 (TMP-d9) were purchased from Sigma-Aldrich (St. Louis, MO, USA).

Stock solutions were prepared at a concentration of 1000 mg/L of each compound by exactly weighing and dissolving in their suitable solvent solution and were stored at −18 °C for 6 months. The tetracyclines, macrolides, sulfonamides and diaminopyrimidines were dissolved in methanol. Fluoro (quinolones) in alkalized methanol (1 M sodium hydroxide (99:1, *v*/*v*)). The working stock solutions mixture (1 µg/mL) and the internal standard solutions mixture—IS (2 µg/mL) were prepared by dilution in ultrapure water and stored at <8 °C.

### 3.3. Sample Preparation

The soil sample pretreatment step was based on drying the sample at 25–30 °C/24 h in a laboratory incubator (Advantage Lab, Darmstadt, Germany) and then sieved through a metal sieve with a mesh diameter of 0.5 mm. Two grams of soil were weighed into 50 mL centrifuge tubes then 50 µL of IS mixture (2 µg/mL) and 1 mL of ultrapure water was added, vortexed, and stored in a dark place at room temperature for 10 min. Then, 8 mL of acetonitrile (vortex) and 0.5 mL of 10% citric acid pH = 4.0 were added and mixed on a rotary stirrer for one hour, followed by ultrasonication for 30 min at room temperature. Subsequently, samples were centrifuged at 3060 × RFC for 10 min at 4 °C. The supernatant was transferred into Oasis HLB cartridges (using as a filter), and filtrated supernatant was collected in a glass tube and evaporated to dryness under a stream of N_2_ at 45 ± 5 °C. The dry residue was dissolved in 500 µL of 0.025% HFBA and put into LC vials.

### 3.4. Final UHPLC-MS-MS Setup and Parameters

Chromatographic analyses were performed using a Shimadzu ultra-high-performance liquid chromatography (UHPLC) Nexera X2 system (Shimadzu, Kyoto, Japan) connected to the QTRAP^®^ 4500 mass spectrometer (Sciex Framingham, MA, USA).

Separation was achieved by using a Luna^®^ Omega 1.6 µm Polar C18 10 column (100 × 2.1 mm, Phenomenex, Torrance, CA, USA) integrated with a guard column of the same type, placed in a column oven at a temperature of 35 °C and with an operating flow rate of 0.4 mL/min. The gradient started with 92% mobile phase B (0.025% HFBA) and 8% mobile phase A (acetonitrile) holding for 30 sec and decreased to 20% within 2.30 min and then decreased to 10% within the next 1 min. This composition was held for 1 min, and again increased to 92% mobile phase B and held for 2 min. Following this gradient, the whole separation was fulfilled within 7 min. The injection volume was 5 μL.

Mass spectrometry measurement was achieved using QTRAP^®^ 4500 triple quadrupole mass spectrometer (Sciex Framingham, MA, USA) operating in positive electrospray ionization (ESI+) mode with the following adjusted parameters: IonSpray voltage: 5500 V, source temperature of 470 °C, curtain Gas—20 psi, ion source gas 1–50 psi, ion source gas 2–60 psi. Fragmentation of the analytes was induced under multiple reaction monitoring mode (MRM). The specific ion transitions and parameters for each compound are listed in Table 1. Instrument control and data processing were provided by the Analyst Software version 1.6.3.

### 3.5. Method Validation

The developed method was validated according to the Commission Implementing Regulation (EU) 2021/808 of 22 March 2021 document [37], regarding the term of linearity, precision (repeatability and within-laboratory reproducibility), selectivity, recovery, ruggedness and matrix effect. The limit of quantification (LOQ) and limit of detection (LOD) were measured based on “Guidance Document on the Estimation of LOD and LOQ for Measurements in the Field of Contaminants in Feed and Food EUR 28099 EN” [47].

Linearity was evaluated by performing a matrix-matched calibration curve prepared by spiking antibiotic-free soil samples, using eight concentration levels (LOQ, 50, 75, 100, 150, 200, 250, and 300 µg/kg). The first point of matrix-matched calibration curve was 1, 5, 10, or 20 µg/kg, depending on the analyte (Table 2). Selectivity was established by analysing 6 different blank soil samples to test for potential interference with endogenous substances. Repeatability was evaluated by fortifying 6 blank samples at three concentration levels: LOQ; 1.5 × LOQ and 2 × LOQ, depending on analytes and were analysed by the same operator on the same day with the same instrument. The within-laboratory reproducibility was established by analysing two additional series of blank samples at the same concentration levels for repeatability, which was analysed by different operators with the same instrument on two different days. Coefficients of variation CVs (%) were calculated. Recovery was evaluated in the same experiment as repeatability by dividing the mean measured concentration by a particular fortification level. The LOD was calculated as the signal-to-noise ratio (S/N) ≈ 3. The LOQ of the method was set as the lowest point of the calibration curve for which the coefficient of variation (CV) was acceptable. The ruggedness of the method was evaluated by introducing minor changes in the sample preparation step. The following parameters were tested in the calculation of ruggedness of the method: the temperature of supernatant evaporation (40 vs. 45 °C), time of vortexing (30 vs. 60 s), the volume of organic extraction solvent (8 vs. 10 mL) and different brands of SPE cartridges; samples were prepared by two different analysts in two different days. The matrix effect (ME, %) was investigated by comparing six spiked samples with standards in solvent (water) at the corresponding concentration of 50 µg/kg It was considered that for values of ME% = 100% ± 15%, this effect was not observed

## 4. Conclusions

In this study, an affordable, rugged and simple UHPLC-MS/MS method for quantifying the concentration of 29 antimicrobial compounds from various classes in soil samples was developed. The application of LLE followed by filtration with SPE cartridges, without the need for a conditioning stage, proved to be a quick and easy step for sample preparation. One of the main advantages of the developed method is the possibility to simultaneously determine five antimicrobial compound classes (tetracyclines, fluoroquinolones, macrolides, sulfonamides and diaminopirymidines). This method showed satisfactory results in linearity, precision, selectivity, recovery, ruggedness and matrix effect for all analytes. it was successfully used in the analysis of veterinary antimicrobial agents in various soil samples collected from different European countries. The results obtained in this study can help understand and improve the knowledge of environmental AMR.

## Figures and Tables

**Table 1:** Summary of the MRM monitored for analytes and MS/MS parameters.

Group	Analyte	Ion Transition 1 [*m*/*z*]	Ion Transition 2 [*m*/*z*]	Retention Time (min)	DP [V]	CE [eV]
Tetracyclines	OTC	461/426	461/444	2.85	50	28
	TC	445/410	445/427	2.93	55	27
	CTC	479/444	479/462	3.09	50	28
	DC	445/428	445/154	2.94	60	23
	DMC(IS)	465/448	-	2.83	48	25
(Fluoro) quinolones	CIP	332/314	332/231	2.85	65	28
	ENR	360/342	360/286	2.93	100	33
	DIF	400/382	400/356	3.04	50	30
	DAN	358/340	358/255	2.86	60	33
	FLU	262/244	262/202	3.63	44	25
	MAR	363/345	363/320	2.42	70	30
	SAR	385/368	385/348	2.72	50	31
	NOR	320/302	320/231	2.82	50	30
	OXO	262/244	262/216	3.29	53	25
	NAL	233/215	233/187	3.61	42	30
	CIP-d8(IS)	340/322	-	2.89	68	29
Macrolides	ERY	734/576	734/158	3.25	75	28
	TYL	916/ 174	916/772	3.26	110	51
	TLM	806/577	806/230	2.92	61	33
	TIL	869/696	869/174	2.83	135	56
	JOS	828/173	828/229	3.59	80	46
	SPI	843/540	843/174	2.98	120	44
	AZY	749/591	749/158	3.01	89	53
Sulfonamides	SME	265/156	265/108	2.90	40	25
	SMT	279/156	279/108	3.20	50	25
	SDMX	311/156	311/108	3.34	50	23
	SMA	254/107	254/155	2.79	42	24
	SMM	281/156	281/108	3.01	50	35
	SFT	256/156	256/108	2.65	53	20
	SDZ	251/156	251/108	2.64	53	22
	SFF(IS)	315/156	-	3.37	90	26
Diaminopirimidines	TMP	292/262	292/231	2.85	52	36
	TMP-d9(IS)	300/234	-	2.90	55	32

*m*/*z*—mass-to-charge ratio; DP—declustering potential; CE—collision energy.

**Table 2:** Validation results.

Analyte	Repeatability *, (CV, %)	Within-Lab Reproducibility *, (CV, %)	LOQ [µg/kg]	LOD [µg/kg]	Recovery * (%)	Matrix Effect [%]
DC	7.3 ± 0.7	10.0 ± 0.9	5.0	0.5	101 ± 1.6	93.5 ± 0.9
OTC	11.3 ± 1.3	7.5 ± 0.8	10.0	1.0	109 ± 1.8	91.7 ± 1.3
TC	1.1 ± 0.8	9.3 ± 1.2	5.0	0.5	99.4 ± 1.1	104 ± 0.5
CTC	14.6 ± 1.4	7.8 ± 1.3	10.0	1.0	106.8 ± 1.0	86.7 ± 0.4
CIP	9.3 ± 0.8	5.9 ± 0.6	20.0	2.0	104 ± 1.0	91.1 ± 1.3
ENR	10.1 ± 1.1	14.1 ± 1.5	10.0	1.0	102 ± 1.9	93.6 ± 0.8
DIF	3.9 ± 0.7	5.1 ± 0.4	20.0	2.0	109 ± 1.5	87.7 ± 0.2
DAN	12.2 ± 1.5	6.7 ± 0.6	10.0	1.0	107 ± 1.4	92.6 ± 0.5
FLU	2.5 ± 0.4	6.3 ± 0.6	5.0	0.5	96.7 ± 1.6	89.7 ± 0.8
MAR	2.1 ± 0.4	9.3 ± 1.2	20.0	2.0	110 ± 1.3	93.4 ± 0.7
NAL	6.1 ± 0.9	7.9 ± 0.9	5.0	0.5	99.7 ± 1.4	117 ± 1.2
OXO	9.9 ±1.2	8.5 ± 0.5	5.0	0.5	99.6 ± 0.8	112 ± 0.3
SAR	5.8 ± 0.8	9.7 ± 1.1	20.0	2.0	102 ± 1.0	86.5 ± 0.6
NOR	10.2 ± 1.3	9.4 ± 1.4	20.0	2.0	100 ± 1.0	94.8 ± 1.1
ERY	6.8 ± 0.8	14.2 ± 1.6	10.0	1.0	89.2 ± 0.8	83.5 ± 1.3
TYL	8.5 ± 0.4	10.3 ± 0.8	5.0	0.5	100 ± 1.4	86.4 ± 1.7
TIL	3.8 ± 0.7	12.7 ± 1.3	5.0	0.5	95.4 ± 1.2	82.6 ± 1.8
JOS	13.4 ± 1.6	10.0 ± 1.4	5.0	1.0	106 ± 1.0	88.1 ± 1.1
SPI	4.9 ± 0.2	10.0 ± 0.8	10.0	1.0	105 ± 1.1	87.3 ± 0 4
TLM	12.2 ± 1.3	8.7 ± 0.8	20.0	2.0	97.4 ± 1.8	92.4 ± 0.9
AZY	1.4 ± 0.4	14.8 ± 1.8	1.0	0.1	90.4 ± 1.2	96.4 ± 1.0
SMT	3.0 ± 0.4	10.1 ± 0.5	5.0	0.5	91.3 ± 0.8	99.8 ± 0.4
SME	8.30 ± 1.0	4.30 ± 0.4	5.0	0.5	100 ± 1.5	101 ± 0.8
SDMX	3.10 ± 0.5	9.30 ± 0.6	5.0	0.5	95.3 ± 1.4	93.4 ± 0.7
SMA	5.8 ± 0.5	10.4 ± 0.4	5.0	0.5	108 ± 1.3	92.6 ± 1.0
SMM	11.6 ± 1.1	8.8 ± 1.3	5.0	0.5	99.2 ± 1.0	95.4 ± 1.3
SFT	4.8 ± 0.4	5.7 ± 0.8	5.0	0.5	107 ± 1.0	98.8 ± 0.8
SMP	9.4 ± 0.7	7.6 ± 0.6	5.0	0.5	93.1 ± 1.1	93.5 ± 1.6
SDZ	1.6 ± 0.3	6.0 ± 0.7	5.0	0.5	95.6 ± 1.3	92.4 ± 1.2
TMP	11.8 ± 1.2	7.6 ± 1.3	10.0	1.0	113 ± 1.7	118 ± 0.7

* measured for LOQ level.

**Table 3:** Sampling area description.

Country	Sampling Area	Type of Soil	Type of Fertilization	Sampling Site
Austria	The Hydrological Open Air Laboratory (HOAL) is situated in Petzenkirchen	Cambisols Planosols Gleysols	natural fertilizers (swine manure) artificial fertilizers (calcium ammonium nitrate)	Crops (wheat, corn) Forest (control) Meadow (control)
Czech Republic	Conventional agricultural land	Cambisols	natural fertilizers (swine and cow manure)	Crops (wheat, oilseed rape) Forest (control) Meadow (control)
Estonia	Conventional agricultural land	Loam soils	natural fertilizers (swine and cow manure) artificial fertilizers	Crops (wheat) Forest (control) Meadow (control)
Portugal	The Portuguese Open Air Laboratory (OAL)	Loamic Calcaric Cambisol Gleyic Fluvisol	natural fertilizers (swine manure)	Crops (mix of oats and vetch) Forest (control) Meadow (control)

## Data Availability

All data are contained within the article.

## References

[1] World Organisation for Animal Health (2019). Annual Report on Antimicrobial Agents Intended for Use in Animals 7th Report.

[2] European Medicines Agency (2020). Sales of Veterinary Antimicrobial Agents in 31 European Countries in 2018—10th ESVAC Report.

[3] Wei R., Ge F., Zhang L., Hou X., Cao Y., Gong L., Chen M., Wang R., Bao E. (2016). Occurrence of 13 veterinary drugs in animal manure-amended soils in Eastern China. Chemosphere.

[4] Robles-Jimenez L.E., Aranda-Aguirre E., Castelan-Ortega O.A., Shettino-Bermudez B.S., Ortiz-Salinas R., Miranda M., Li X., Angeles-Hernandez J.C., Vargas-Bello-pérez E., Gonzalez-Ronquillo M. (2022). Worldwide traceability of antibiotic residues from livestock in wastewater and soil: A systematic review. Animals.

[5] Eurostat Statistics|Eurostat. https://ec.europa.eu/eurostat/databrowser/view/tps00202/default/map?lang=en.

[6] (2019). EC Regulation (EU) 1069/2009 Animal By-Products. Off. J. Eur. Union.

[7] Zubair M., Wang S., Zhang P., Ye J., Liang J., Nabi M., Zhou Z., Tao X., Chen N., Sun K. (2020). Biological nutrient removal and recovery from solid and liquid livestock manure: Recent advance and perspective. Bioresour. Technol..

[8] Vaneeckhaute C., Meers E., Michels E., Buysse J., Tack F.M.G. (2013). Ecological and economic benefits of the application of bio-based mineral fertilizers in modern agriculture. Biomass Bioenergy.

[9] Li J., Xin Z., Zhang Y., Chen J., Yan J., Li H., Hu H. (2017). Long-term manure application increased the levels of antibiotics and antibiotic resistance genes in a greenhouse soil. Appl. Soil Ecol..

[10] Li C., Chen J., Wang J., Ma Z., Han P., Luan Y., Lu A. (2015). Occurrence of antibiotics in soils and manures from greenhouse vegetable production bases of Beijing, China and an associated risk assessment. Sci. Total Environ..

[11] Karci A., Balcioǧlu I.A. (2009). Investigation of the tetracycline, sulfonamide, and fluoroquinolone antimicrobial compounds in animal manure and agricultural soils in Turkey. Sci. Total Environ..

[12] Li Y.W., Wu X.L., Mo C.H., Tai Y.P., Huang X.P., Xiang L. (2011). Investigation of sulfonamide, tetracycline, and quinolone antibiotics in vegetable farmland soil in the pearl river delta area, Southern China. J. Agric. Food Chem..

[13] Kim J.W., Hong Y.K., Ryu S.H., Kwon O.K., Lee Y.B., Kim S.C. (2023). Development of analytical method for veterinary antibiotics and monitoring of residuals in agricultural environment. Appl. Biol. Chem..

[14] Qasim B., Razzak A.A., Motelica-Heino M., Kamil G.M., Morabito D. (2020). Quantitative determination of fluoroquinolones in contaminated soils by HPLC with solid-phase extraction. Baghdad Sci. J..

[15] Cycoń M., Mrozik A., Piotrowska-Seget Z. (2019). Antibiotics in the soil environment—Degradation and their impact on microbial activity and diversity. Front. Microbiol..

[16] Boxall A.B.A., Johnson P., Smith E.J., Sinclair C.J., Stutt E., Levy L.S. (2006). Uptake of veterinary medicines from soils into plants. J. Agric. Food Chem..

[17] Tasho R.P., Cho J.Y. (2016). Veterinary antibiotics in animal waste, its distribution in soil and uptake by plants: A review. Sci. Total Environ..

[18] Madikizela L.M., Ncube S., Chimuka L. (2018). Uptake of pharmaceuticals by plants grown under hydroponic conditions and natural occurring plant species: A review. Sci. Total Environ..

[19] Zhang H., Zhou Y., Huang Y., Wu L., Liu X., Luo Y. (2016). Residues and risks of veterinary antibiotics in protected vegetable soils following application of different manures. Chemosphere.

[20] Jechalke S., Heuer H., Siemens J., Amelung W., Smalla K. (2014). Fate and effects of veterinary antibiotics in soil. Trends Microbiol..

[21] Aga D.S., O’Connor S., Ensley S., Payero J.O., Snow D., Tarkalson D. (2005). Determination of the persistence of tetracycline antibiotics and their degradates in manure-amended soil using enzyme-linked immunosorbent assay and liquid chromatography-mass spectrometry. J. Agric. Food Chem..

[22] Hamscher G., Pawelzick H.T., Höper H., Nau H. (2005). Different behavior of tetracyclines and sulfonamides in sandy soils after repeated fertilization with liquid manure. Environ. Toxicol. Chem..

[23] Grenni P., Ancona V., Barra Caracciolo A. (2018). Ecological effects of antibiotics on natural ecosystems: A review. Microchem. J..

[24] Sonola V.S., Katakweba A.S., Misinzo G., Matee M.I.N. (2021). Occurrence of Multi-Drug-Resistant *Escherichia coli* in Chickens, Humans, Rodents and Household Soil in Karatu, Northern Tanzania. Antibiotics.

[25] Bengtsson-Palme J. (2017). Antibiotic resistance in the food supply chain: Where can sequencing and metagenomics aid risk assessment?. Curr. Opin. Food Sci..

[26] Fang H., Han Y., Yin Y., Pan X., Yu Y. (2014). Variations in dissipation rate, microbial function and antibiotic resistance due to repeated introductions of manure containing sulfadiazine and chlortetracycline to soil. Chemosphere.

[27] Góchez D., Raicek M., Ferreira J.P., Jeannin M., Moulin G., Erlacher-Vindel E. (2019). OIE annual report on antimicrobial agents intended for use in animals: Methods used. Front. Vet. Sci..

[28] Graham D.W., Bergeron G., Bourassa M.W., Dickson J., Gomes F., Howe A., Kahn L.H., Morley P.S., Scott H.M., Simjee S. (2019). Complexities in understanding antimicrobial resistance across domesticated animal, human, and environmental systems. Ann. N. Y. Acad. Sci..

[29] Samreen, Ahmad I., Malak H.A., Abulreesh H.H. (2021). Environmental antimicrobial resistance and its drivers: A potential threat to public health. J. Glob. Antimicrob. Resist..

[30] Kumar Mehata A., Lakshmi Suseela M.N., Gokul P., Kumar Malik A., Kasi Viswanadh M., Singh C., Selvin J., Muthu M.S. (2022). Fast and highly efficient liquid chromatographic methods for qualification and quantification of antibiotic residues from environmental waste. Microchem. J..

[31] Chan C.L., Wai H.K.F., Wu P., Lai S.W., Chan O.S.K., Tun H.M. (2022). A Universal LC-MS/MS Method for Simultaneous Detection of Antibiotic Residues in Animal and Environmental Samples. Antibiotics.

[32] Kokoszka K., Kobus A., Bajkacz S. (2021). Optimization of a method for extraction and determination of residues of selected antimicrobials in soil and plant samples using HPLC-UV-MS/MS. Int. J. Environ. Res. Public Health.

[33] Schlüsener M.P., Spiteller M., Bester K. (2003). Determination of antibiotics from soil by pressurized liquid extraction and liquid chromatography-tandem mass spectrometry. J. Chromatogr. A.

[34] Hoff R., Pizzolato T.M., Diaz-Cruz M.S. (2016). Trends in sulfonamides and their by-products analysis in environmental samples using mass spectrometry techniques. Trends Environ. Anal. Chem..

[35] Huygens J., Rasschaert G., Heyndrickx M., Dewulf J., Van Coillie E., Quataert P., Daeseleire E., Becue I. (2022). Impact of fertilization with pig or calf slurry on antibiotic residues and resistance genes in the soil. Sci. Total Environ..

[36] Mishra A., Chhonker Y.S., Bisen A.C., Prasad Y.D., Tulsankar S.L., Chandasana H., Dey T., Verma S.K., Bala V., Kanojiya S. (2020). Rapid and Simultaneous Analysis of Multiple Classes of Antimicrobial Drugs by Liquid Chromatography-Tandem Mass Spectrometry and Its Application to Routine Biomedical, Food, and Soil Analyses. ACS Omega.

[37] Chen G., Li M., Liu X. (2015). Fluoroquinolone Antibacterial Agent Contaminants in Soil/Groundwater: A Literature Review of Sources, Fate, and Occurrence. Water. Air. Soil Pollut..

[38] Franklin A.M., Andrews D.M., Williams C.F., Watson J.E. (2022). Simultaneous Extraction of Four Antibiotic Compounds from Soil and Water Matrices. Separations.

[39] Gbylik-Sikorska M., Posyniak A., Mitrowska K., Gajda A., Błądek T., Şniegocki T., Zmudzki J. (2014). Occurrence of veterinary antibiotics and chemotherapeutics in fresh water, sediment, and fish of the rivers and lakes in Poland. Bull. Vet. Inst. Pulawy.

[40] Chopra I., Roberts M. (2001). Tetracycline Antibiotics: Mode of Action, Applications, Molecular Biology, and Epidemiology of Bacterial Resistance. Microbiol. Mol. Biol. Rev..

[41] Askari Rizvi S.F. (2018). Tetracycline: Classification, Structure Activity Relationship and Mechanism of Action as a Theranostic Agent for Infectious Lesions-A Mini Review. Biomed. J. Sci. Tech. Res..

[42] Commission E. (2021). Commission Implementing Regulation (EU) 2021/808 of 22 March 2021 on the performance of analytical methods for residues of pharmacologically active substances used in food-producing animals and on the interpretation of results as well as on the methods to. Off. J. Eur. Union.

[43] Holmes N.E., Charles P.G.P. (2009). Safety and Efficacy Review of Doxycycline. Clin. Med. Ther..

[44] Nahler G., Nahler G. (2009). Committee for Veterinary Medicinal Products (CVMP). Dict. Pharm. Med..

[45] EMA (2019). Categorisation of antibiotics for use in animals for prudent and responsible use. Eur. Med. Agency.

[46] SUNU WHO List of Critically Important Antimicrobials for Human Medicine (WHO CIA List). http://who.int/foodsafety/publications/antimicrobials-fifth/en/.

[47] Wenzl T., Haedrich J., Schaechtele A., Robouch P., Stroka J. (2016). Guidance Document on the Estimation of LOD and LOQ for Measurements in the Field of Contaminants in Feed and Food 2016.

